# Effect of High Hydrostatic Pressure Processing on Microbiological Shelf-Life and Quality of Fruits Pretreated with Ascorbic Acid or SnCl_2_

**DOI:** 10.1155/2014/819209

**Published:** 2014-09-11

**Authors:** Anthoula A. Argyri, Chrysoula C. Tassou, Fotios Samaras, Constantinos Mallidis, Nikos Chorianopoulos

**Affiliations:** Hellenic Agricultural Organization (DEMETER), Institute of Technology of Agricultural Products, Sof. Venizelou 1, Lycovrissi, 14123 Athens, Greece

## Abstract

In the current study, the processing conditions required for the inactivation of *Paenibacillus polymyxa* and relevant spoilage microorganisms by high hydrostatic pressure (HHP) treatment on apricot, peach, and pear pieces in sucrose (22°Brix) solution were assessed. Accordingly, the shelf-life was determined by evaluating both the microbiological quality and the sensory characteristics (taste, odor, color, and texture) during refrigerated storage after HHP treatment. The microbiological shelf-life of apricots, peaches, and pears was prolonged in the HHP-treated products in comparison with the untreated ones. In all HHP-treated packages for apricots, peaches, and pears, all populations were below the detection limit of the method (1 log CFU/g) and no growth of microorganisms was observed until the end of storage. Overall, no differences of the L*, a*, or b* value among the untreated and the HHP-treated fruit products were observed up to the time at which the unpressurized product was characterized as spoiled. HHP treatment had no remarkable effect on the firmness of the apricots, peaches, and pears. With regard to the sensory assessment, the panelists marked better scores to HHP-treated products compared to their respective controls, according to taste and total evaluation during storage of fruit products.

## 1. Introduction

High hydrostatic pressure (HHP) processing is a novel nonthermal method which has revealed great potential in producing microbiologically safer products while maintaining the natural characteristics of the food products [1]. The use of HHP in food preservation has been acknowledged as an alternative method to thermal processes [2]. HHP practical application in the food industry has taken place in the past twenty years [3–5], usually for a range of pressures between 100 and 800 MPa.

One of the principal advantages of the HHP method is the substantial increase in shelf-life and improvement of food safety due to the inactivation affected in the microbial population [1, 5]. HHP destroys vegetative cells and inactivates certain enzymes [6] with an insignificant change in the sensory characteristics [7, 8]. The resistance of microorganisms is variable, depending on the type of organism and the food matrix involved [1]. Spores show higher resistance to inactivation by HHP.* Bacillus* spp. form high-pressure resistant spores and they have been suggested to be used as the target organism in the development of standards for HHP treatments [9, 10]. There are few reports on the behavior of HHP-treated* Bacillus* spp. in foods [11, 12], whereas in all cases a combination of HHP and mild temperature had to be used to accomplish a noteworthy loss of viability.

During the last years, processed fruit products have become well known due to the increased demand of consumers for healthy diets. Fresh-cut fruits have occurred as common snacks in food services [13]. Overall, they have a short shelf-life because of enzymatic browning, tissue softening, and microbial growth [14]. The use of the HHP treatment in such products is an effective tool to enhance the microbiological safety and shelf-life of various types of fruit products and juices [15, 16].

A number of various methods may be used to control the browning reactions in fruit products and extend their shelf-life. In this respect, the most effective process is the heat treatment, but it leads to changes in quality characteristics of the fruit products that are not friendly to the consumer. Browning can also be delayed by the elimination of oxygen and by the use of oxygen-impermeable packaging [14]. An alternative approach to control browning is through the use of additives such as ascorbic acid or SnCl_2_, which may act by reducing the formed o-quinones back to o-diphenolic compounds, avoiding thus the existence of the secondary pigment formation [14]. Undesirably, when ascorbic acid is oxidized, the o-quinones can amass with browning reactions [17].

In the present study, HHP treatment was applied to preserve fresh-cut apricot, peach, and pear pieces in sucrose solution. To our knowledge, there is limited, if any, information relevant to the potential use of HHP treatment in fresh-cut apricot, peach, or pear pieces preservation. Consequently, the process conditions toward inactivation of* Paenibacillus polymyxa* (a microorganism relevant to the spoilage of fresh-cut fruit pieces) by HHP were optimized for the first time. A shelf-life study of apricot, peach, and pear pieces in sucrose solution HHP treated was assessed, where color, texture, and sensory evaluation were monitored in parallel with the microbiological control during refrigerated storage at 5°C.

## 2. Materials and Methods

### 2.1. Preparation of the Apricot, Peach, and Pear Pieces

For the series of experiments, apricots (cultivar Bebekou), peaches (cultivar Andross), and pears (cultivar Williams) were bought in a local market and thoroughly washed with water and 70% ethanol. Consequently, apricots and peaches were dipped for 30 seconds in boiling water containing 2% NaOH to loosen skins, dipped quickly in cold water, and peeled. Then the fruits were cut in half with a sterile knife and the pits were removed. The pear fruits were peeled, cut in half with a sterile knife and the core was removed. Finally, all fruits were cut with a sterile knife into approximately 0.5 cm thick cube pieces.

### 2.2. Production of* Paenibacillus polymyxa* Spores

The strain used in this study was the* Paenibacillus polymyxa* DSM 36 (DSMZ, Germany). The strain was revived from a stock culture stored at −80°C, by subculturing twice in 10 mL nutrient broth (LAB014, LAB M), incubated for 48 h at 30°C. The fresh cultures were heat shocked (80°C for 10 min) prior to inoculation on agar plates to allow uniform sporulation. Subsequently, the above suspension was spread on nutrient agar (LAB008, LAB M) plates containing 0,06 g/L MgSO_4_ *και* 0,25 g/L K_2_HPO_4_ (pH adjusted at 7.0) (NA-MK) and incubated for approximately 7 days at 30°C to allow time for the cells to sporulate. When at least 90% cells have sporulated (evaluated by contrast phase microscopy), the spores were harvested by depositing 1 mL of sterile water onto each NA-MK plate and transferred into a sterile centrifugation tube. The spore suspension was centrifuged at 4000 ×g for 20 min at 4°C and the pellet was rinsed with ice-cold distilled water. The latter procedure was repeated four times. The final pellet was resuspended in a small volume of ice-cold distilled water and stored at 4°C until use.

### 2.3. Inoculation of the Samples

The prepared apricot, peach, and pear cuts were inoculated with spores of* P. polymyxa* to reach a final concentration of 400 CFU/g on the fruits. A part of the fruit samples was not inoculated with* P. polymyxa* to serve as samples for the sensory analyses. The inoculated or not samples were subsequently treated with the following preprocessing handlings.

### 2.4. Preprocessing of the Samples Prior to the HHP Treatment

The fruit preparation as well as the following 1st and 2nd handling was performed according to different processing procedures followed in canned food industry for each fruit type. The 3rd handling was followed to evaluate the effect of HHP processing on the cut fruits without additional thermal treatment.

*1st Handling*. 100 g of cut-fruit pieces was dipped in hot water (*T* > 95°C) for 2 min, subsequently transferred to sterile polyethylene bags (180 mm × 300 mm, film thickness 90 *μ*m, FlexoPack) and 100 mL of boiling-hot sucrose solution (22.7% sucrose/22°Brix) was added. The average final temperature of the product was 81°C.

*2nd Handling*. 100 g of cut-fruit pieces was washed in cold water, subsequently transferred to sterile polyethylene bags (180 mm × 300 mm, film thickness 90 *μ*m, FlexoPack) and 100 mL of boiling-hot sucrose solution (22.7% sucrose/22°Brix) was added. The average final temperature of the product was 61°C.

*3rd Handling*. 100 g of cut-fruit pieces was washed in cold water, subsequently transferred to sterile polyethylene bags (180 mm × 300 mm, film thickness 90 *μ*m, FlexoPack) and 100 mL of sucrose solution (*T* = 30°C) (22.7% sucrose/22°Brix) was added. The final temperature of the product was 25°C.

The handlings 1, 2, and 3 were applied to apricots and peaches, whereas handlings 1 and 3 were applied to pears. In half packages with apricots and peaches, SnCl_2_ (30 ppm) was added, while in half packages with pears ascorbic acid (0.1%) was added. Then, the pouches were heat-sealed after careful removal of air, moved directly to the HHP unit, and treated in a single run at 600 MPa for 5 min at 10°C, as described below. Additionally, samples treated according to 1st handling without any additive and HHP treatment served as control samples. The HHP-treated and control samples were subsequently stored at 5°C. Each experiment was replicated three times (three different batches of each fruit).

### 2.5. High Hydrostatic Pressure (HHP) Treatment

HHP inactivation experiments were conducted in triplicate at pressure of 600 MPa and temperature 10°C for processing time of 5 minutes. The high pressure unit (Food Pressure Unit FPU 1.01, Resato International BV, Roden, Holland) comprised of a pressure intensifier and a multivessel system consisting of a central vessel of 250 mL capacity, with a maximum operating pressure and temperature of 1000 MPa and 90°C. The pressure transmitting fluid used was polyglycol ISO viscosity class VG 15 (Resato International BV, Roden, Holland). Process temperature in the vessel was achieved by liquid circulation in the outer jacket controlled by a heating cooling system [18, 19]. The desired value of pressure was set and after pressure build up (20 MPa/s) the pressure vessels were isolated. The pressure of the vessel was released after a preset time interval (according to the experimental design) by opening the corresponding pressure valve [18, 19]. The initial adiabatic temperature increase during pressure build up was taken into consideration in order to achieve the desired operating temperature during pressurization. Pressure and temperature were constantly monitored and recorded (in 1 s intervals) during the process [18, 19]. The come-up rate was approximately 100 MPa per 7 sec and the pressure release time was 3 sec. Pressurization time reported in this work does not include the pressure come-up and release times.

### 2.6. Microbiological Analysis

Samples were analyzed throughout storage at regular time intervals during storage of apricot (0, 103, 166, 231, and 287 days), peach (0, 48, 104, 170, and 226 days), and pear (0, 67, 122, and 185 days). Fruit pieces were weighed aseptically, added to sterile 1/4 strength Ringer's solution, and homogenized in a stomacher (Lab Blender 400, Seward Medical, London, UK) for 60 s at room temperature. Decimal dilutions in 1/4 strength Ringer's solution were prepared and duplicate 1 or 0.1 mL aliquots of appropriate dilutions was pour- or spread-plated on the following media: (i) plate count agar (CM0325, Oxoid) for total viable counts, incubated at 30°C for 48–72 h; (ii) de Man-Rogosa-Sharp (MRS) medium (CM 0361, Oxoid) for lactic acid bacteria (LAB), adjusted in pH 5.7, overlaid with the same medium, and incubated at 30°C for 48–72 h; (iii) tryptone dextrose extract agar (CM0075, Oxoid) containing 0.1% activated carbon; (iv) rose Bengal chloramphenicol agar base (LAB 36 supplemented with selective supplement X009, LAB M), for yeasts/molds incubated at 25°C for 72 h. In all growth media incubation time was extended by 1-2 days to allow recovery of lethally/sublethally injured or stressed by HHP treatment cells.

### 2.7. Firmness Measurement

The firmness of the fruit pieces was determined using a TA.HD* plus* texture analyser equipped with a Kramer shear cell (Stable Microsystems, Surrey, UK) with the following test parameters: load cell = 500 kg, test speed = 5 mm/s, Krammer shear cell (Stable Microsystems, Surrey, UK). The fruit cuts of a total weight of ca. 20 g were placed on the Kramer cell. The procedure was followed at least 4 times for each case. The speed setting was 200 mm/sec, whereas the penetration force was measured in N. The values were recorded and firmness was calculated dividing the maximum shear compression force by the total weight of fruits and expressed as N/g of product.

### 2.8. Color Measurement

Color change was measured using a Minolta Chroma Meter fitted with a CR-300 measuring head (Minolta, Osaka, Japan). The apparatus was calibrated with a standard white tile (*X* = 78.66, *Y* = 83.31, and *Z* = 88.40). The recorded values *X*, *Y*, and *Z* were converted to CIE L*, a*, and b* color values. The L* value indicates the visual lightness or the luminance on a scale of 0 to 100 (0 = perfect black, 100 = perfect white). Positive a* values indicate red direction; negative a* value is the green direction. Positive b* values are the yellow direction, and negative b* values are the blue direction. At each sampling time, 5 random measurements of the fruits of each different treatment were performed from duplicate samples, from 3 different batches of each fruit.

### 2.9. Sensory Analysis

For the sensory evaluation, taste and total evaluation (color, texture, taste, and odor) of the noninoculated products were assessed from a panel of eight members (staff from the laboratory). The same trained persons were used in each evaluation, and all were blinded to which sample was being tested. The sensory evaluation was carried out in artificial light and the temperature of packaged product was similar to ambient temperature. Assessment was designed to identify spoilage conditions exclusively. A persistent dull appearance, or unusual color or appearance, was considered unacceptable. Taste was scored on a three-point hedonic scale where 0 = good, 1 = acceptable, and 2 = totally unacceptable. Scores above 1 rendered the product spoiled and indicated the end of the product's shelf-life. Total evaluation was scored on a ten-point hedonic scale where 10 = very good, 5 = acceptable, and 1 = totally unacceptable. Scores below 5 rendered the product spoiled and indicated the end of the product's shelf-life.

## 3. Results

### 3.1. HHP Inactivation of Microorganisms in Apricot, Peach, and Pear Pieces in Syrup

The growth of spoilage microorganisms and* P. polymyxa* was monitored during refrigerated storage of the apricot, peach, and pear pieces in sucrose solution after HHP treatment at 600 MPa for 10 min. In all HHP-treated packages for apricots (Table 1), peaches (Table 2), and pears (Table 3), no growth of microorganisms was observed, whereas all populations were below the detection limit of the method (1 log CFU/g). In the respective control cases for each fruit (Tables 1, 2, and 3), growth was observed for total viable counts, lactic acid bacteria, and yeasts, and all the control samples were spoiled until the end of storage. The same results were monitored for the inoculated* P. polymyxa*, which was found to be below the detection limit in HHP-treated packages for apricots (Table 1), peaches (Table 2), and pears (Table 3).

### 3.2. Effect of HHP Treatment on the Quality Parameters of Apricot, Peach, and Pear Pieces in Syrup during Storage

An increase was observed in lightness (L*) of apricot samples during storage in all treatments, with changes found to be more intense in control samples (Table 1). a* values were found to increase during storage of control samples, indicating a gradual browning of the samples. Browning was also observed in samples treated according to the 3rd handling with or without SnCl_2_ after the HHP application in comparison with control samples, but no further browning was observed during storage. No browning was observed during storage of samples treated according to 1st or 2nd handling with or without SnCl_2_ (Table 1). Finally, b* values were found to increase especially during storage of control and 3rd handling samples, meaning that there is an increase in samples yellowness (Table 1). A decrease was observed in firmness of apricot cuts of all treatments during storage, except from the 2nd handling cases, where a hardening of apricot cuts was noticed (Table 1). Moreover, the firmness values of samples treated according to the 1st handling decreased earlier in comparison with the rest of handlings. However, the addition of SnCl_2_ showed a positive effect in the firmness of apricot samples treated according to 1st handling. Finally, no changes were observed between the samples within the same treatment of 1st or 3rd handling, with or without the addition of SnCl_2_.

No specific trend was observed in L* or b* values of the peach samples of all treatments during storage (Table 2). However, an increase was monitored in a* values of samples for all cases (browning) with changes being observed earlier in 3rd handling samples without the addition of SnCl2 (Table 2). A decrease was observed in firmness of samples for all treatments during storage. The application of HHP had no effect in the firmness of the samples, since the samples treated with the 3rd handling had similar firmness values with control. However, an additional effect of the thermal processing on reducing the firmness of samples treated according to the 1st and 2nd handling was observed, which was more intense in the case of 1st handling (stronger thermal treatment). Finally, no particular changes were observed between the samples of the same treatment after the addition of SnCl_2._

In pear samples, no remarkable changes were observed in the color parameters. Indicatively, L* values are given in Table 3. It was observed that L* values decreased slightly during storage of samples treated with the 3rd handling with ascorbic acid. Similarly, a* values showed an increase in the latter case only, while b* values showed no particular changes (data not shown). It was observed that, in control samples, the firmness decreased during storage. On the other hand, the application of HHP had as a result a slight decrease in firmness values (3rd handling), with no further reduction observed during storage (Table 3). This effect increased with the previous thermal treatment of the pear cuts (1st handling), with no further reduction during storage being observed in this case as well.

### 3.3. Sensory Analysis

For the sensory evaluation, taste and total evaluation (color, texture, taste, and odor) of the products were assessed from a panel of eight members. The shelf-life of the products treated with the different handlings in comparison with the control is presented in Tables 1, 2, and 3. The panelists marked with better scores the HHP-treated products compared to their respective controls, according to taste and total evaluation during storage of apricots, peaches, and pears (Tables 1, 2, and 3). The addition of SnCl_2_ or ascorbic acid gave similar or higher scoring for total evaluation and taste of the products within the same treatment. More specifically, the scores of the total evaluation were higher during storage of HHP-treated samples of all fruits with the SnCl_2_ or ascorbic acid, while the corresponding taste scores were found to be similar (Tables 1, 2, and 3). In addition, the samples treated with the 1st handling gave better scores in comparison with the rest of handlings (especially the 3rd one) that scores were found to be below the acceptable boundaries. It has to be noted that for all cases of HHP treated fruit samples (irrespective of the fruit type), a more transparent appearance of the fruit cuts was observed by the panelists.

## 4. Discussion

In the current study, the potential of HHP treatment to preserve fresh-cut apricot, peach, and pear pieces in sucrose solution was assessed. Therefore, the processing conditions toward inactivation of* P. polymyxa*—a microorganism relevant to the spoilage of fresh-cut fruit pieces—were studied. In addition, a shelf-life study of HHP-treated apricot, peach, and pear pieces in sucrose solution was evaluated, in which color, texture, and sensory evaluation were assessed in parallel to the microbiological quality during refrigerated storage at 5°C.

In the present work, the syrup in which the apricot, peach, or pear pieces were dipped into did not only provide an acidic environment, but also a high concentration of sucrose. Several authors have observed the protective effect of such solutions on the inactivation of bacteria and yeasts, despite the low *a*_*w*_ [14, 20–22]. Although sucrose solution provided a protected environment on the bacteria and yeast cells, a total reduction of microorganisms (below the detection limit of the method) was achieved with the application of HHP, irrespective of the previous thermal treatment of the fruit cuts. These results are in accordance with other studies relevant to the reduction of microorganisms caused by HHP [14, 23, 24]. In addition, several authors have reported that HHP sensitizes bacteria cells to low pH [25–28], while others have reported that, even after a pressure level of 600 MPa, cells are able to grow in such acidic fruit products during refrigerated storage [29, 30]. Furthermore, the inactivation of the* P. polymyxa* spores after the application of HHP treatment is in accordance with previous reports that a minimum pressure level of 600 MPa is needed for the inactivation of bacteria and molds spores [31–33].

Generally, the presence of SnCl_2_ or ascorbic acid in the HHP-treated samples had no effect on the color parameters within the same treatment. This indicates that either the HHP application gives the maximum effect that can be given or the applied SnCl_2_ or ascorbic acid concentration as indicated from the industry should be elevated to meet the needs of the new processing method. However, the different handlings seemed to have an effect on the color, especially in the browning of apricot (Table 1). It was shown that the application of thermal treatments—a strong one (1st handling) or a milder (2nd handling)—had a positive effect in preventing the fruit browning, most probably due to the thermal inactivation of the enzymes. In contrast, fruit samples treated with the 3rd handling exhibited higher a* values (similar or higher to the control samples), indicating the nessecary thermal treament of fruits cuts, irrespective of the HHP application. It has been reported that, for natural peach puree and peach puree containing ascorbic acid or cysteine and treated with HHP (517 MPa/5 min), an increased color maintenance of HHP-processed purees was observed [34]. In another report [35], the authors managed to prevent browning on HHP-treated apples during storage by using pineapple juice.

The changes in the firmness values of fruit cuts were different according to the fruit type. The application of the HHP alone (3rd handling) had no effect in the firmness of peach and apricot cuts but reduced the firmness of pears. Moreover, the previous heat treatments of the samples (1st and 2nd handling) did not initially affect the firmness of apricots but reduced the firmness of pears and peaches. However, in most of the cases, the HHP application resulted in higher firmness values of the samples during storage, in comparison with the control samples. It has been reported that when the fruits are stored in pouches with syrup, as in the current study, texture degradation may occur due to solubilization and depolymerization of water-soluble pectin sodium carbonate-soluble pectin [36]. The pectin degradation and softening of flesh previously treated with thermal and HHP processing (600 MPa for 5, 10, or 30 min) can be delayed with a storage temperature of 4 ± 1°C [36]. A firming effect of HHP at 600 MPa for 5 min on apple pieces was reported previously [35], while in another study, no significant decrease of hardness was observed during storage of HHP-treated (600 MPa for 10 min) apple pieces, in comparison with the untreated ones that showed a significant decrease [14]. This could be due to the damage of the fruit tissue caused by the high population of the yeasts/molds in the products [14].

The scores of the total sensory evaluation were higher during storage of HHP-treated samples of all fruits with the SnCl_2_ or ascorbic acid, while the corresponding taste scores were found to be similar (Tables 1, 2, and 3). In addition, the samples treated with the 1st handling gave better scores in comparison with the rest of handlings, especially the 3rd one that scores were found to be below the acceptable boundaries. Moreover, for all cases of HHP treated fruit samples (irrespective of the fruit type), the fruit cuts had a more transparent appearance, which is in agreement with other reports [14, 22]. The changes in the nature and overall appearance of the products may be attributed to the damage of the fruit tissues caused by HHP [37].

## 5. Conclusion

The HHP treatment was applied to enhance the microbiological quality and extend the shelf-life of apricot, peach, and pear pieces in sucrose solution. Treatment at 600 MPa for 5 min with the combination of thermal processing of the fruits can remarkably extend the shelf-life of these fresh products, with minor changes on their color and firmness. However, additional research is needed in order to optimize the handling prior to HHP, aiming to better quality and sensory attributes of such products.

## Figures and Tables

**Table 1:** Evolution of microbiological counts (log⁡ CFU/g), firmness, L*, a*, b* values, and sensory evaluation in HHP-treated at 600 MPa for 5 min at 10°C and untreated (control case) apricot pieces in sucrose solution with or without SnCl_2_ during storage (5°C). Each data point is an average ± standard deviation from three individual batches.

	Time (days)						Time (days)				
	0	103	166	231	287		0	103	166	231	287
*P. polymyxa *						L* value					
Control	1.3 ± 0.1	2.1 ± 0.4	4.2 ± 0.7	ND	ND	Control	29.3 ± 0.5	32.5 ± 1.2	37.7 ± 1.4	37.8 ± 2.3	38.5 ± 1.1
1st handling without SnCl_2_	No growth detected (<dl during 287 days)					1st handling without SnCl_2_	29.5 ± 1.7	31.9 ± 2.5	32.4 ± 2.2	32.9 ± 1.1	35.9 ± 1.2
1st handling with SnCl_2_	No growth detected (<dl during 287 days)					1st handling with SnCl_2_	30.4 ± 1.4	33.5 ± 0.8	31.0 ± 1.5	33.9 ± 3.4	34.7 ± 2.1
2nd handling without SnCl_2_	No growth detected (<dl during 287 days)					2nd handling without SnCl_2_	31.2 ± 0.9	33.1 ± 2.6	31.9 ± 0.7	31.8 ± 1.9	34.8 ± 1.4
2nd handling with SnCl_2_	No growth detected (<dl during 287 days)					2nd handling with SnCl_2_	30.5 ± 1.6	31.7 ± 1.2	32.0 ± 2.3	35.0 ± 1.7	35.2 ± 2.0
3rd handling without SnCl_2_	No growth detected (<dl during 287 days)					3rd handling without SnCl_2_	29.9 ± 1.5	31.8 ± 2.3	29.4 ± 0.6	34.6 ± 2.2	31.8 ± 0.8
3rd handling with SnCl_2_	No growth detected (<dl during 287 days)					3rd handling with SnCl_2_	30.1 ± 1.6	30.2 ± 2.2	31.9 ± 1.0	35.8 ± 0.5	35.3 ± 1.9

*Total viable counts *						a* value					
Control	1.7 ± 0.2	5.2 ± 1.3	6.8 ± 0.5	ND	ND	Control	0.2 ± 0.0	0.4 ± 0.1	−0.1 ± 0.0	1.4 ± 0.1	1.3 ± 0.1
1st handling without SnCl_2_	No growth detected (<dl during 287 days)					1st handling without SnCl_2_	0.1 ± 0.0	0.2 ± 0.0	−0.5 ± 0.0	−0.1 ± 0.0	−0.1 ± 0.0
1st handling with SnCl_2_	No growth detected (<dl during 287 days)					1st handling with SnCl_2_	0.4 ± 0.1	0.8 ± 0.1	0.6 ± 0.1	0.1 ± 0.1	0.2 ± 0.0
2nd handling without SnCl_2_	No growth detected (<dl during 287 days)					2nd handling without SnCl_2_	0.3 ± 0.0	0.6 ± 0.1	0.7 ± 0.1	0.3 ± 0.0	−0.1 ± 0.0
2nd handling with SnCl_2_	No growth detected (<dl during 287 days)					2nd handling with SnCl_2_	0.7 ± 0.1	1.1 ± 0.2	1.0 ± 0.1	0.6 ± 0.1	0.1 ± 0.0
3rd handling without SnCl_2_	No growth detected (<dl during 287 days)					3rd handling without SnCl_2_	1.4 ± 0.2	1.5 ± 0.2	1.1 ± 0.0	1.6 ± 0.2	1.2 ± 0.1
3rd handling with SnCl_2_	No growth detected (<dl during 287 days)					3rd handling with SnCl_2_	1.9 ± 0.1	1.8 ± 0.1	2.2 ± 0.2	1.8 ± 0.2	1.2 ± 0.1

*Lactic acid bacteria *						b* value					
Control	<dl	1.4 ± 0.1	2.2 ± 0.3	ND	ND	Control	10.5 ± 0.3	12.6 ± 0.2	14.6 ± 0.3	16.9 ± 0.3	16.5 ± 0.8
1st handling without SnCl_2_	No growth detected (<dl during 287 days)					1st handling without SnCl_2_	10.2 ± 0.2	11.4 ± 0.2	10.2 ± 1.0	10.4 ± 0.2	14.5 ± 0.3
1st handling with SnCl_2_	No growth detected (<dl during 287 days)					1st handling with SnCl_2_	12.1 ± 0.3	13.3 ± 0.4	11.5 ± 0.3	13.1 ± 0.5	13.8 ± 0.6
2nd handling without SnCl_2_	No growth detected (<dl during 287 days)					2nd handling without SnCl_2_	12.0 ± 0.2	13.0 ± 0.4	12.0 ± 0.8	11.4 ± 0.7	13.5 ± 0.5
2nd handling with SnCl_2_	No growth detected (<dl during 287 days)					2nd handling with SnCl_2_	11.9 ± 0.1	12.3 ± 0.2	11.9 ± 0.9	14.6 ± 0.8	13.8 ± 0.2
3rd handling without SnCl_2_	No growth detected (<dl during 287 days)					3rd handling without SnCl_2_	12.4 ± 0.2	13.2 ± 0.1	10.7 ± 0.7	14.7 ± 0.5	10.7 ± 0.3
3rd handling with SnCl_2_	No growth detected (<dl during 287 days)					3rd handling with SnCl_2_	11.2 ± 0.1	11.9 ± 0.4	14.0 ± 0.2	16.4 ± 1.4	14.2 ± 0.6

*Yeasts *						Taste^a^					
Control	<dl	4.9 ± 0.7	6.7 ± 0.4	ND	ND	Control	0.0	1.4	2.0	ND	ND
1st handling without SnCl_2_	No growth detected (<dl during 287 days)					1st handling without SnCl_2_	0.0	0.3	0.8	0.5	0.4
1st handling with SnCl_2_	No growth detected (<dl during 287 days)					1st handling with SnCl_2_	0.0	0.1	0.9	0.3	0.7
2nd handling without SnCl_2_	No growth detected (<dl during 287 days)					2nd handling without SnCl_2_	0.0	0.4	0.9	1.3	0.5
2nd handling with SnCl_2_	No growth detected (<dl during 287 days)					2nd handling with SnCl_2_	0.0	0.8	1.0	0.9	0.5
3rd handling without SnCl_2_	No growth detected (<dl during 287 days)					3rd handling without SnCl_2_	0.0	1.0	1.4	1.1	1.3
3rd handling with SnCl_2_	No growth detected (<dl during 287 days)					3rd handling with SnCl_2_	0.1	0.9	1.4	0.9	0.9

*Firmness *						Total evaluation^b^					
Control	34.0 ± 2.4	20.5 ± 1.2	15.8 ± 2.2	24.5 ± 3.5	15.0 ± 2.3	Control	10.0	3.8	2.8	ND	ND
1st handling without SnCl_2_	34.1 ± 1.7	29.6 ± 3.3	14.9 ± 1.1	18.0 ± 2.2	13.5 ± 2.5	1st handling without SnCl_2_	10.0	6.3	6.4	6.6	4.9
1st handling with SnCl_2_	34.1 ± 1.7	33.6 ± 2.8	25.4 ± 2.5	22.5 ± 1.4	18.0 ± 3.1	1st handling with SnCl_2_	10.0	7.8	6.4	8.4	4.6
2nd handling without SnCl_2_	32.4 ± 3.0	49.9 ± 2.5	38.5 ± 3.0	40.5 ± 3.2	43.0 ± 2.6	2nd handling without SnCl_2_	10.0	7.4	3.9	3.1	6.7
2nd handling with SnCl_2_	32.4 ± 3.0	42.2 ± 3.7	37.8 ± 2.3	34.8 ± 2.0	36.5 ± 2.9	2nd handling with SnCl_2_	10.0	5.3	4.9	6.0	7.8
3rd handling without SnCl_2_	35.0 ± 2.3	33.3 ± 3.2	35.9 ± 1.7	30.0 ± 2.6	31.0 ± 1.7	3rd handling without SnCl_2_	10.0	4.0	4.0	3.8	2.4
3rd handling with SnCl_2_	35.0 ± 2.3	32.1 ± 2.4	24.9 ± 2.1	29.0 ± 4.4	30.0 ± 2.8	3rd handling with SnCl_2_	10.0	6.0	4.0	5.5	6.2

ND: not determined-spoiled product, dl: detection limit (1 log⁡ CFU/g); ^a^the taste is the mean value of scores given by 8 panelists-0: good, 1: acceptable, 2: spoiled; ^b^the total evaluation is the mean value of odor, color, texture, and taste of 8 panelists. -10: very good, 5: acceptable, and 1: totally unacceptable.

**Table 2:** Evolution of microbiological counts (log⁡ CFU/g), firmness, L*, a*, b* values, and sensory evaluation in HHP-treated at 600 MPa for 5 min at 10°C and untreated (control case) peach pieces in sucrose solution with or without SnCl_2_ during storage (5°C). Each data point is an average ± standard deviation from three individual batches.

	Time (days)						Time (days)				
	0	48	104	170	226		0	48	104	170	226
*P. polymyxa *						L* value					
Control	1.5 ± 0.2	3.1 ± 0.5	5.2 ± 0.6	ND	ND	Control	39.8 ± 2.3	40.4 ± 1.1	40.9 ± 3.4	41.8 ± 1.9	40.0 ± 1.7
1st handling without SnCl_2_	No growth detected (<dl during 226 days)					1st handling without SnCl_2_	39.5 ± 1.6	36.9 ± 2.5	40.0 ± 2.2	42.2 ± 1.2	39.7 ± 2.5
1st handling with SnCl_2_	No growth detected (<dl during 226 days)					1st handling with SnCl_2_	38.4 ± 1.4	39.5 ± 0.8	39.7 ± 1.5	38.5 ± 0.5	38.2 ± 1.7
2nd handling without SnCl_2_	No growth detected (<dl during 226 days)					2nd handling without SnCl_2_	37.2 ± 0.9	35.7 ± 2.0	39.9 ± 0.8	40.7 ± 1.1	41.4 ± 1.4
2nd handling with SnCl_2_	No growth detected (<dl during 226 days)					2nd handling with SnCl_2_	40.5 ± 1.6	42.3 ± 1.2	43.3 ± 2.3	40.3 ± 1.5	39.8 ± 2.0
3rd handling without SnCl_2_	No growth detected (<dl during 226 days)					3rd handling without SnCl_2_	39.9 ± 1.5	40.8 ± 2.3	40.4 ± 0.6	41.1 ± 2.2	37.0 ± 1.9
3rd handling with SnCl_2_	No growth detected (<dl during 226 days)					3rd handling with SnCl_2_	40.1 ± 1.6	40.2 ± 2.2	42.0 ± 1.6	40.3 ± 2.3	40.2 ± 1.6

*Total viable counts *						a* value					
Control	1.8 ± 0.1	5.4 ± 0.8	6.5 ± 0.4	ND	ND	Control	−3.2 ± 0.0	−3.0 ± 0.1	−2.3 ± 0.0	−2.7 ± 0.1	−2.4 ± 0.2
1st handling without SnCl_2_	No growth detected (<dl during 226 days)					1st handling without SnCl_2_	−2.7 ± 0.0	−1.9 ± 0.0	−2.7 ± 0.0	−3.0 ± 0.0	−1.6 ± 0.1
1st handling with SnCl_2_	No growth detected (<dl during 226 days)					1st handling with SnCl_2_	−3.0 ± 0.1	−2.8 ± 0.1	−2.3 ± 0.1	−2.5 ± 0.0	−1.1 ± 0.1
2nd handling without SnCl_2_	No growth detected (<dl during 226 days)					2nd handling without SnCl_2_	−2.9 ± 0.0	−2.2 ± 0.2	−2.8 ± 0.0	−2.8 ± 0.0	−1.1 ± 0.1
2nd handling with SnCl_2_	No growth detected (<dl during 226 days)					2nd handling with SnCl_2_	−3.7 ± 0.1	−3.4 ± 0.2	−3.8 ± 0.1	−2.4 ± 0.0	−1.6 ± 0.0
3rd handling without SnCl_2_	No growth detected (<dl during 226 days)					3rd handling without SnCl_2_	−3.4 ± 0.2	−3.0 ± 0.2	−1.9 ± 0.0	−1.5 ± 0.5	−1.9 ± 0.1
3rd handling with SnCl_2_	No growth detected (<dl during 226 days)					3rd handling with SnCl_2_	−3.9 ± 0.1	−3.2 ± 0.1	−2.2 ± 0.2	−2.9 ± 0.1	−1.3 ± 0.1

*Lactic Acid Bacteria *						b* value					
Control	<dl	1.1 ± 0.1	2.2 ± 0.2	ND	ND	Control	17.8 ± 0.3	17.9 ± 0.3	18.0 ± 0.2	18.1 ± 0.2	17.9 ± 0.1
1st handling without SnCl_2_	No growth detected (<dl during 226 days)					1st handling without SnCl_2_	17.2 ± 0.2	18.0 ± 0.2	16.3 ± 1.0	18.1 ± 0.2	15.9 ± 0.4
1st handling with SnCl_2_	No growth detected (<dl during 226 days)					1st handling with SnCl_2_	17.1 ± 0.3	16.9 ± 0.4	17.4 ± 0.3	16.9 ± 0.3	13.2 ± 0.3
2nd handling without SnCl_2_	No growth detected (<dl during 226 days)					2nd handling without SnCl_2_	18.0 ± 0.2	17.6 ± 0.4	18.0 ± 0.8	18.7 ± 0.3	16.6 ± 0.5
2nd handling with SnCl_2_	No growth detected (<dl during 226 days)					2nd handling with SnCl_2_	19.9 ± 0.1	19.3 ± 0.2	19.9 ± 0.9	17.8 ± 0.8	17.1 ± 0.6
3rd handling without SnCl_2_	No growth detected (<dl during 226 days)					3rd handling without SnCl_2_	18.4 ± 0.3	18.8 ± 0.1	18.8 ± 0.7	18.6 ± 0.1	16.0 ± 0.4
3rd handling with SnCl_2_	No growth detected (<dl during 226 days)					3rd handling with SnCl_2_	18.2 ± 0.1	18.3 ± 0.4	19.3 ± 0.2	18.1 ± 0.4	17.0 ± 0.2

*Yeasts *						Taste^a^					
Control	<dl	3.8 ± 0.8	6.3 ± 0.7	ND	ND	Control	0.0	1.3	2.0	ND	ND
1st handling without SnCl_2_	No growth detected (<dl during 226 days)					1st handling without SnCl_2_	0.0	0.4	0.9	1.1	1.2
1st handling with SnCl_2_	No growth detected (<dl during 226 days)					1st handling with SnCl_2_	0.0	0.2	0.7	0.9	0.8
2nd handling without SnCl_2_	No growth detected (<dl during 226 days)					2nd handling without SnCl_2_	0.1	0.5	0.9	1.2	1.5
2nd handling with SnCl_2_	No growth detected (<dl during 226 days)					2nd handling with SnCl_2_	0.0	0.6	1.0	1.1	0.9
3rd handling without SnCl_2_	No growth detected (<dl during 226 days)					3rd handling without SnCl_2_	0.0	1.1	1.3	1.2	1.6
3rd handling with SnCl_2_	No growth detected (<dl during 226 days)					3rd handling with SnCl_2_	0.1	0.8	1.2	1.1	1.3

*Firmness *						Total evaluation^b^					
Control	104.1 ± 1.0	94.1 ± 1.7	92.6 ± 2.4	82.4 ± 3.2	85.0 ± 2.3	Control	10.0	3.7	2.9	ND	ND
1st handling without SnCl_2_	73.0 ± 2.3	53.6 ± 2.8	52.6 ± 2.2	48.0 ± 3.7	35.5 ± 3.2	1st handling without SnCl_2_	10.0	6.5	6.0	6.3	4.6
1st handling with SnCl_2_	73.0 ± 2.3	55.4 ± 2.5	49.0 ± 3.5	54.5 ± 2.3	31.9 ± 1.7	1st handling with SnCl_2_	10.0	7.9	6.8	6.4	4.9
2nd handling without SnCl_2_	91.0 ± 2.2	72.5 ± 1.4	68.2 ± 3.2	81.5 ± 2.0	69.0 ± 2.6	2nd handling without SnCl_2_	10.0	7.1	3.8	3.3	4.7
2nd handling with SnCl_2_	91.0 ± 2.2	68.0 ± 3.0	49.0 ± 2.6	73.0 ± 2.9	69.0 ± 1.7	2nd handling with SnCl_2_	10.0	6.3	4.7	5.0	5.8
3rd handling without SnCl_2_	103.0 ± 1.9	70.5 ± 1.2	63.8 ± 2.8	77.5 ± 3.5	84.5 ± 2.1	3rd handling without SnCl_2_	10.0	4.2	3.6	3.3	2.8
3rd handling with SnCl_2_	103.1 ± 1.9	85.8 ± 2.2	80.2 ± 2.4	84.5 ± 2.3	84.0 ± 4.4	3rd handling with SnCl_2_	10.0	7.0	5.0	5.9	5.2

ND: not determined-spoiled product, dl: detection limit (1 log⁡ CFU/g); ^a^the taste is the mean value of scores given by 8 panelists-0: good, 1: acceptable, 2: spoiled, ^b^the total evaluation is the mean value of odor, color, texture, and taste of 8 panelists. -10: very good, 5: acceptable, and 1: totally unacceptable.

**Table 3:** Evolution of microbiological counts (log⁡ CFU/g), firmness, L* values, and sensory evaluation in HHP-treated at 600 MPa for 5 min at 10°C and untreated (control case) pear pieces in sucrose solution with or without ascorbic acid during storage (5°C). Each data point is an average ± standard deviation from three individual batches.

	Time (days)						Time (days)		
	0	67	122	185		0	67	122	185
*P. polymyxa *					*Firmness *				
Control	1.2 ± 0.2	2.4 ± 0.2	4.7 ± 0.5	ND	Control	141.0 ± 12.5	70.5 ± 5.0	67.0 ± 8.0	57.0 ± 6.5
1st handling without ascorbic acid	No growth detected (<dl during 185 days)				1st handling without ascorbic acid	75.5 ± 7.5	101.0 ± 8.0	96.0 ± 6.5	92.5 ± 9.5
1st handling with ascorbic acid	No growth detected (<dl during 185 days)				1st handling with ascorbic acid	75.5 ± 7.5	73.5 ± 5.5	78.5 ± 7.5	79.5 ± 7.0
3rd handling without ascorbic acid	No growth detected (<dl during 185 days)				3rd handling without ascorbic acid	100.0 ± 10.5	116.5 ± 6.0	122.5 ± 10.5	116.5 ± 11.5
3rd handling with ascorbic acid	No growth detected (<dl during 185 days)				3rd handling with ascorbic acid	100.0 ± 10.5	107.0 ± 9.5	116.5 ± 11.0	133.0 ± 12.5

*Total Viable Counts *					L* value				
Control	1.4 ± 0.2	4.3 ± 0.4	6.9 ± 0.7	ND	Control	39.5 ± 3.3	40.1 ± 4.0	38.6 ± 3.0	39.6 ± 2.4
1st handling without ascorbic acid	No growth detected (<dl during 185 days)				1st handling without ascorbic acid	38.8 ± 2.4	38.9 ± 2.6	40.3 ± 2.5	38.4 ± 1.0
1st handling with ascorbic acid	No growth detected (<dl during 185 days)				1st handling with ascorbic acid	37.4 ± 2.5	37.1 ± 3.1	40.0 ± 4.2	34.8 ± 3.7
3rd handling without ascorbic acid	No growth detected (<dl during 185 days)				3rd handling without ascorbic acid	36.9 ± 3.1	36.1 ± 2.4	37.8 ± 1.7	37.4 ± 2.2
3rd handling with ascorbic acid	No growth detected (<dl during 185 days)				3rd handling with ascorbic acid	37.1 ± 1.6	36.9 ± 2.0	34.7 ± 3.2	31.4 ± 2.8

*Lactic acid bacteria *					Taste^a^				
Control	<dl	1.2 ± 0.1	2.0 ± 0.4	ND	Control	0.0	1.2	2.0	ND
1st handling without ascorbic acid	No growth detected (<dl during 185 days)				1st handling without ascorbic acid	0.0	0.4	0.5	0.5
1st handling with ascorbic acid	No growth detected (<dl during 185 days)				1st handling with ascorbic acid	0.1	0.3	0.3	0.8
3rd handling without ascorbic acid	No growth detected (<dl during 185 days)				3rd handling without ascorbic acid	0.1	1.4	1.4	1.4
3rd handling with ascorbic acid	No growth detected (<dl during 185 days)				3rd handling with ascorbic acid	0.0	0.8	0.6	0.8

*Yeasts *					Total Evaluation^b^				
Control	<dl	4.1 ± 0.5	6.3 ± 0.6	ND	Control	10.0	6.4	3.1	ND
1st handling without ascorbic acid	No growth detected (<dl during 185 days)				1st handling without ascorbic acid	10.0	8.0	4.6	5.6
1st handling with ascorbic acid	No growth detected (<dl during 185 days)				1st handling with ascorbic acid	10.0	8.5	8.3	7.9
3rd handling without ascorbic acid	No growth detected (<dl during 185 days)				3rd handling without ascorbic acid	10.0	3.0	4.0	3.5
3rd handling with ascorbic acid	No growth detected (<dl during 185 days)				3rd handling with ascorbic acid	10.0	5.5	5.9	5.8

ND: not determined-spoiled product, dl: detection limit (1 log⁡ CFU/g); ^a^the taste is the mean value of scores given by 8 panelists-0: good, 1: acceptable, 2: spoiled; ^b^the total evaluation is the mean value of odor, color, texture, and taste of 8 panelists. -10: very good, 5: acceptable, and 1: totally unacceptable.
